# The Nursing of Our Soldiers.—A Serious Difficulty

**Published:** 1900-01-13

**Authors:** 


### The Institutional Workshop.

### THE NURSING OF OUR SOLDIERS.—A SERIOUS DIFFICULTY.

In consequence of the liberal offers from various quarters to provide fully-equipped hospitals to work in conjunction with, and subordinate to the military authorities in South Africa, a most important question arises in regard to the personnel of such establishments. An edict has gone forth that female nurses are to be employed at the base hospitals only, and that all other hospitals, not merely those in the immediate rear of the combatant forces, but those on the lines of communication also, are to be nursed, as is the case in the purely military hospitals, by men only. Now here comes a great difficulty. The military hospitals are nursed by men of the Army Medical Corps, men who have been trained in their duties and have had considerable experience. The base hospitals have trained female nurses, and so also have what we may call the auxiliary hospitals, so long as they are at the base. But if an auxiliary hospital is to leave the base, and occupy a position along the 500 and odd miles of the so-called lines of communication, men only can be employed. The working of this regulation has, then, this sinister consequence, that unless the auxiliary hospitals can find male nurses they are tied to the base, or, in other words, to the coast. Unfortunately, there are no male nurses, and if an auxiliary hospital wants to move forward it must get men together as it can—St. John Ambulance men probably, good men and well trained in first aid, but not practically trained or experienced in nursing.

But is there any valid reason why female nurses should be restricted to the base hospitals alone? If our lines of communication lay in the enemy's country, and if our base were at our frontier, there might be some reason in the arrangement. But for hundreds of miles they are within our own settled territory, and what we are now treating as our base is the coast 500 miles from the scene of action, a 500 miles of country inhabited by our own people—men, women, and children—occupying farms and villages and towns, and enjoying the ordinary attributes of civilisation. Is this district so wild, so uncivilised, so dangerous, that nurses cannot properly be sent there? We think not. It is a serious matter, and it loooks as if much were being sacrificed to mere War Office red-tape. So long as trained nursing is confined to the base, serious surgical treatment must be to a large extent restricted to the base. But is there any excuse for base hospitals being so far off? For a long distance inland the country is perfectly safe, and is covered with farms and villages and townships, and it would seem only reasonable that some of the hospitals on these lines of communication should be nursed by trained women nurses, who can be obtained in any quantity.

The urgency of some relaxation of this rule about women at the base only is illustrated by the following extract from a letter from Cape Town in the *Guy's Hospital Gazette,* in regard to the transit of the wounded after the battle of Belmont:—

"As many patients were put into the train as the train could conceivably hold, and the passage was littered up with men in stretchers or men sitting about or lying on the floor. Consequently it was absolutely hopeless for the R.A.M.C. captain, the civil surgeon, and the two nursing sisters to interfere in any way with the wounds. It was as much as they could do to administer nourishment and stimulants, and to keep the dust off the patients' faces. The line all along was cleared for the hospital train, and thus the 591 miles with four or five stoppages were accomplished in 32 hours. But, admitting the merits of this organisation, one must also be deeply impressed with the terrors and dangers that these 32 hours on the railway must have imposed upon the 103 wounded travellers. The 103, with certain exceptions, were the most severely wounded that had survived and were considered capable of being further treated—compound fractures of the thigh of the most horrible description, bullet wounds of the skull with extensive fracture of vault or base, perforated lungs, fractured pelvis, and even bullet wounds of the abdomen with perforation of the intestine. Is it justifiable, we keep asking ourselves, to subject these terribly serious cases to the strain of such a journey? Of course, the answer to this is strategical rather than surgical, otherwise one would say with the grumblers, who are many, that we ought to have been stationed at De Aar instead of Wynberg."

